# Comparative Analysis of the Influence of Selected Physical Factors on the Level of Pain in the Course of Temporomandibular Joint Disorders

**DOI:** 10.1155/2020/1036306

**Published:** 2020-10-10

**Authors:** Łukasz Kopacz, Żaneta Ciosek, Helena Gronwald, Piotr Skomro, Roman Ardan, Danuta Lietz-Kijak

**Affiliations:** ^1^Department of Propaedeutic, Physical Diagnostics and Dental Physiotherapy, Faculty of Medicine and Dentistry, Pomeranian Medical University, Szczecin, Poland; ^2^Department of Medical Rehabilitation and Clinical Physiotherapy, Pomeranian Medical University, Szczecin, Poland; ^3^Department of Econometrics, Faculty of Economic Sciences, Koszalin University of Technology, Koszalin, Poland

## Abstract

**Introduction:**

Temporomandibular joints (TMJs) play a very significant function in the activity of the locomotor system of the masticatory system. But they are often a source of pain, myopathy, myoarthropathy, and malfunction of their surrounding or internal structures. The treatment of a patient's discomfort associated with masticatory system dysfunctions strongly depends on their cause. *Aim of the Study*. The objective of the study was to evaluate the impact of selected physical factors: LED light therapy with electromagnetic field and cryotherapy for the level of pain, in the treatment of patients suffering from temporomandibular disorders (TMDs).

**Materials and Methods:**

The study included 60 patients of both genders with diagnosed TMD in a clinical trial. The participants were randomly divided into two groups. Each group consisted of 30 people and was subjected to separate therapies in which LED light therapy with electromagnetic field (MLT) and cryotherapy (CT) were applied.

**Results:**

Having assessed the results of the author's own research in terms of analgesic activity, determined on the VAS scale during the daily routine activity of the mandible and its individual movements, in general, each of the studied groups demonstrated a considerable decrease in the level of the patients' perception of pain (<0.001). Having compared both the therapeutic methods used, a greater reduction in the level of perceived pain was achieved with MLT (*p*=0.002). The type of therapy used turned out to be the only significant factor for the magnitude of this reduction.

**Conclusions:**

Conclusions based on the results of our own research indicate that the selected methods of treatment demonstrate an analgesic effect in terms of the overall discomfort in the course of TMD, and that they may be an alternative pain relief thereby reducing the patient's intake of painkillers.

## 1. Introduction

One of the main symptoms of a dysfunction within the masticatory system, in addition to functional disorders, is pain, and it is one of the most common reasons for patients to seek help from specialists. This discomfort may be caused by the dysfunction of the masticatory muscles, tendons, or temporomandibular joints (TMJs). In addition, it may occur not only at rest but also during the movement of the mandible [1]. Increased muscle strain on components of the masticatory system can lead to a compensatory rise in tension in adjacent muscle groups. As a result, pain also occurs in the area of the head, neck, neck muscles, spine, and shoulder girdle, even including the lower parts of the musculoskeletal system [2]. Additional symptoms accompanying temporomandibular dysfunctions (TMDs) are pathological tooth wear following bruxism, tinnitus, and a change in psychological profile [3]. Women are more often affected than men [4]. Further signs of dysfunction within the stomatognathic system are restrictions and disorders of mandible mobility, often associated with disabilities and pain while chewing, yawning, or biting hard foods [5]. In addition, we should pay attention to muscular tenderness on palpation and touch, as their hypertrophy is characterised by a “square” facial appearance and migraine headaches [6].

The application of various forms of physiotherapy procedures in dentistry significantly complement the process of treating patients with the abovementioned symptoms, with postoperative complications, as well as those with severe disorders within the locomotor system of the masticatory system. It is primarily aimed at relieving pain, reducing muscle tension within the stomatognathic system, inducing regenerative processes in soft tissue, and restoring the positive psychophysical effect of the patient. The basis of this treatment is the application of the same physiotherapeutic factors during applicable periods, which facilitates the mechanisms of homeostasis equalisation and thus contributes to the stimulation of self-healing reactions of the body. The above-described influence on the human body and its potent development contributed to the fact that dental physiotherapy has become a separate branch of medicine that includes physiotherapy and kinesitherapy, as well as massage [7]. Any physical stimulus may affect tissue, systems, and the entire human body, leading to reduced inflammation, improved circulation, enhanced immunity, and decreased or increased excitability of nerves. Such an action improves the effects of treatment and accelerates the regeneration of the affected tissue and its recovery to physiological efficiency. Moreover, analgesic effects in the area under treatment, as well as the degenerative effects of pathological tissue, are incredibly significant [8, 9]. Frequently, among patients with TMD, specific healing treatments are used, such as electrotherapy, thermotherapy, or phototherapy. However, it should be noted that each type of treatment is a supportive therapy and should not be performed individually, although, in the case of stomatognathic disorders in combination with splint therapy, various methods of physiotherapy or pharmacotherapy are possible [7].

In TMD, the most commonly used physical factors are ozone, a laser with a suitable wavelength, and a slowly alternating electromagnetic field. Studies conducted in the last twenty years prove that combined therapy in the form of LED light therapy (MLT), which is a fusion of the action of the light emitted from high-energy LEDs and a slow alternating extremely low-frequency magnetic field (ELF-MF), produces synergistic therapeutic effects [10]. An electromagnetic field is characterised by its permeability through any structure of the body, and its action may be profound and equal. The appropriate length of light applied simultaneously penetrates deeper than would otherwise be the case with an independent agent. Such an impact of the magnetic field and light is a characteristic of this procedure as other physical factors reach only superficial depth of the tissue. The definition of the treatment with the use of ELF-MF depends on its parameters, and its range allows for the division of procedures into magnetotherapy and magnetostimulation [11]. The emission of electromagnetic radiation through LEDs takes place in the range of red (R, red), infrared (IR, infrared), and mixed light (RIR). The optical radiation energy, in the visible and infrared spectra produced by LEDs, ultimately causes tissue regeneration [12, 13].

The application of another healing stimulus, through low temperatures, is also used in TMD therapies. The influence of cryotherapy on the human body is based primarily on analgesic processes, which promote the treatment of symptom disorders within the stomatognathic system. These effects are caused by lowering the speed of nerve fibre conduction, slowing down the nociceptive conductivity located in the skin tissue and reducing the release of pain mediators. A further impact of cryotherapy beneficially affecting the human body is the inhibition of inflammatory processes, by reducing the level of the metabolism of cells involved in inflammation, as well as decreasing the frequency of enzymatic reactions. The result is a drop in the amount of inflammatory mediators and improved blood supply to the tissues in the affected area [14]. Cryotherapy, in addition to the analgesic effect, has a positive influence on enhanced muscle tension, decreasing it.

Another desirable effect of this procedure is the antioedematous function, as well as inhibiting haemorrhage tendencies. However, during the selection of a cold treatment, and determining the purpose of its action, it should be noted that there is a two-phase vascular reaction to extremely low temperatures [15, 16]. It is also essential that by the consensual reflexes, vascular reactions caused by the cold factor, occurring in the target may also arise in spaces distant from the area of the performed surgery [17, 18].

The local cryotherapy procedure is based on the application of cold within the area subjected to the treatment. The temperature value depends on the agent used during the treatment. The cryogen may be liquid nitrogen, carbon dioxide, or cooled air [19, 20]. Due to the fact that the body's reaction to the stimulus generated during the procedure depends on many factors (e.g., area undergoing the treatment, physiology and type of tissue, gender, age, patient's condition, and type of disease), the therapy should be planned individually. The temperature and the amount of refrigerant required for the treatment, the duration of the procedure, as well as the area undergoing therapy must be taken into account. Despite the existing guidelines for the parameters used in individual diseases, it has been scientifically proved that there is a correlation between changes in the body and the number of treatments performed in a particular series [21].

The main purpose of applying physical stimuli in the course of TMD is primarily to eliminate pain, regulate the metabolism, and improve circulation in overloaded muscles by relaxing them. Although there are a large number of methods used in the course of the described condition, there is no specific and effective one that would present an advantage over other types of therapy. This statement encourages the maintenance of the continuity of research on developing an appropriate method. There are also many trials in which individual therapies are combined to improve the effectiveness of the conducted treatment. The interest in applying cold for therapeutic purposes is still increasing, and the research related to this subject should be constantly developed [22].

## 2. Objective

The study was aimed at examining the effects of selected physical factors, such as cryotherapy (CT) and LED light therapy with electromagnetic field (MLT), on the level of pain experienced by the patient in the course of TMD and comparing their analgesic effects.

## 3. Materials and Methods

The research was conducted at the Department of Propaedeutic, Physical Diagnostics and Dental Physiotherapy of the Pomeranian Medical University in Szczecin. All patients were informed about the aim and course of the study and expressed their written consent for participation. The criteria for inclusion in the study group were the studies based on the diagnostic criteria for temporomandibular disorders —DC/TMD. The research was approved by the Bioethics Committee of the Pomeranian Medical University (Resolution No. KB-0012/36/15 of 23.03.2015 and Annex No. KB-0012/47/17 of 27.02.2017). Participation in the study was voluntary, and during the performance of the research, the anonymity of patients was maintained in accordance with the Act on Personal Data Protection of 29.08.1997 (Journal of Laws No. 133, item 883).

The study group consisted of 60 patients with TMD disorders of a myofascial nature. They were observed and diagnosed in the clinical examination. The examination was performed by a dentist and a physiotherapist. The examined patients were randomly divided into two groups, each represented by 30 patients. The first group treated with LED light therapy accompanied by electromagnetic field (MLT) consisted of 23 women and 7 men, while the other group, undergoing cryotherapy (CT), consisted of 16 women and 14 men. All therapeutic series were carried out in line with the valid and standard procedure in this field. The complete duration of treatment with both physical agents was three weeks with weekend breaks, during which 15 physical therapy treatments were performed. All the patients had a painful form of dysfunction of the masticatory system that manifested itself during daily routine activities. In the clinical trial, from both the groups, those patients were additionally selected who reported pain during specific movements of the mandible: abduction, laterotrusion to the right and left, and protrusion. In the studied patients, pain was assessed before the start of a series of treatments and immediately after the end of the last treatment. For this purpose, the Visual Analogue Scale (VAS) was used, in which the patient determined the degree of discomfort intensity on a 10 cm point scale, where “0” would indicate no pain and “10” would indicate the sharpest pain they can imagine. Values in the VAS 0–3 range indicate correct results of the therapy and a feeling of improvement. However, VAS above 7 means a very severe pain and the necessity for further diagnosis and consultation with a doctor. The study assessed pain during the daily activities of the jaw such as biting, chewing, and yawning. Pain was also assessed during specific movements. The pain level experienced by each patient was assessed before the therapy and immediately after the last treatment from a series of all the procedures undertaken. Nominal measurements (pain/no pain) were made before and after the therapy.

**Figure 1:**
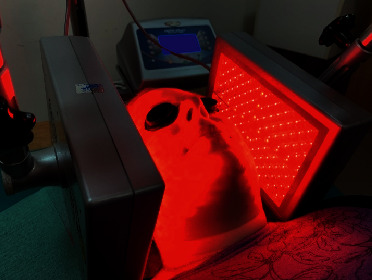
MLT treatment using LED panels (author's photo).

For the purpose of carrying out MLT, a Viofor JPS (Med & Life) device was used, which emits an alternating low-frequency electromagnetic field and light in the visible and invisible range through high-energy LEDs (red light 640 nm and infrared light 830 nm). During therapy, the patient was situated in a comfortable lying position, and the two panels of the device, each containing 280 diodes, were placed parallel to the treated surface at the height of the TMJ area, in direct contact (Figure 1). Out of concern for the patients' safety and with reference to the methodology of the procedure, the patients were asked to clean their face prior to the start of the therapy. The physiotherapist verified the absence of skin lesions in the treated area and provided the patients with safety glasses used at the time of the procedure. The therapy lasted 10 minutes, during which the participant was advised to relax and have a rest. The MLT physical therapy was carried out using an electromagnetic field generated by the system using the М1 method, P3 programme, at an intensity of 6, which ensures the constant application of the selected intensity and applies the highest values of ionic cyclotron resonance generated in cells. Therefore, the frequency of basic pulses was in the range 180–195 Hz, the frequency of pulse packets was in the range 12.5–29 Hz, the groups of packets were in the range of 2.8–7.6 Hz, and the series were in the range of 0.08–0.3 Hz.

**Figure 2:**
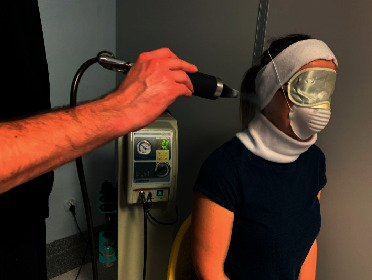
Local cryotherapy procedure in the TMJ area (author's photo).

The local cryotherapy procedure for the TMJ region was performed once a day, using a cryostimulator (CryoFlex, Poland, Cryo-T), operating on the CO_2_ system. The process involves the application of cold air on the area of joints on the right side and left side at a temperature of approximately −70°C at the nozzle's exit. Here, it only triggers a local effect. The type of coolant used for the treatment was carbon dioxide. TMJ was cooled down by a special nozzle directed to the treatment site. The affected space was completely dry, and there were no skin lesions in this particular area. The respiratory tract, lymph nodes, as well as the eyes and ears located within the TMJ, were covered by the patient's face mask, a special band for the eyes, ears, and neck, and the application of earplugs (Figure 2). The nozzle's exit was held at an appropriate distance from the patient's body (at least 10 cm), and the movement of the cryoapplicator nozzle was adjusted in cooperation with the participant to prevent the cold stream from constantly falling on one area of the treatment site. The duration of the procedure was 2 minutes.

Descriptive statistics for quantitative characteristics are presented in terms of mean, standard deviation, and maximum and minimum values. The assumption of the normal distribution of quantitative traits was checked with the Shapiro–Wilk test. In the case of no normal distribution, the Wilcoxon test was applied to compare mean pain intensity values before and after the therapy. In addition, McNemar's chi-square test was used to analyse the nominal data. The results were considered statistically significant at *p* < 0.05. For calculations, *R* package was selected.

## 4. Results

Following the treatment in each group, a decrease in the level of pain during the daily activity of the mandible, such as biting, chewing, and yawning, was observed. The measurements displayed significant deviations from normal distribution; the Wilcoxon test was implemented. The differences before and after therapy were statistically significant (Table 1).

**Figure 3:**
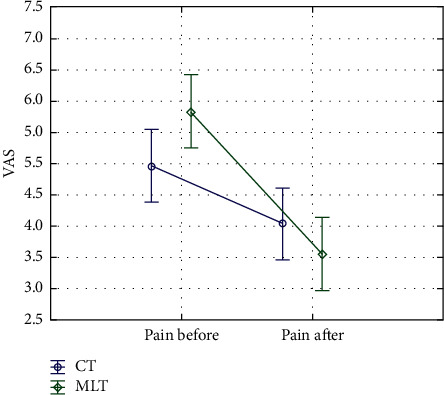
Changes in mean values in the VAS scale before and after the therapy (95% confidence interval for the mean value). ^*∗*^MLT, LED light therapy with electromagnetic field; CT, cryotherapy.

The efficacy of the abovementioned methods was compared using the Mann–Whitney *U* test, which demonstrated a statistically significant difference between methods tested (*p*=0.002^*∗*^). A plot in Figure 3, presenting a change in mean values in the VAS scale, suggests a greater reduction in the level of pain using MLT.

Since the groups were not identical in terms of sex and age, a linear regression model with three independent variables was elaborated: type of therapy, sex (women-0 and men-1), and age (years). Only the type of therapy turned out to be a significant variable (Table 2).

The results of measuring the level of pain during specific movements of the mandible were obtained. A decreasing tendency of pain during the exercise of particular mandibular movements was observed. Upon using the chi-square McNemar test, all the cases yielded statistically significant differences in the number of patients feeling pain.

Our findings demonstrate a large decrease in complaints during the abduction movement in the CT group where pain before and after the treatment was observed in 21 and 7 subjects, respectively. Likewise, after MLT, the number of patients with pain decreased from 18 to 6 (*p* < 0.01 for both the treatments).

Pain during right side laterotrusive movement decreased after applying selected physical factors in the studied groups. Before CT procedures, pain was reported by 14 patients, while after the therapy, by only 4 patients. In the case of MLT, 10 patients reported pain before the procedures and only 3 after treatment (*p* < 0.01 for both the treatments).

Comparing the effect of the applied therapeutic methods in terms of the change of parameters determining the occurrence of pain during the left side laterotrusive movement, a decreasing trend was also observed. Pain was experienced by 15 subjects before the cryotherapy, and 5 subjects after the cryotherapy. In the case of MLT, 8 subjects felt pain discomfort before the treatment, after which this changed to 4 patients (*p* < 0.01 for CT and *p*=0.046 for MLT).

After the therapy in both groups, a reduction in pain during protrusion movement was also observed. In the group covered by CT, 14 patients felt pain during movement before the procedures and only 4 after the procedures. In the case of MLT, 9 patients reported pain discomfort before treatment and 3 after the procedures (*p* < 0.01 for CT and *p*=0.014 for MLT).

## 5. Discussion

The authors' study confirmed the analgesic effect of the selected physical therapy methods. They focused on the analysis of results obtained for each group, in which the influence of individual methods on the level of pain (measured on the VAS scale) during active chewing movements were compared. After the therapy, a decrease in the level of pain during active mandibular movement was noted in each of the studied groups, whereas in general comparison, the differences between both therapies were statistically significant in favour of MLT. There are many publications related to the anti-inflammatory and analgesic effects of the slow alternating low-frequency electromagnetic field. Thomas used this field in his research to treat chronic musculoskeletal pain, providing an analgesic effect. The result obtained in the group of 17 patients was similar to the statistical significance (*p*=0.06) [23]. Calderhead confirmed in his studies that the light radiation energy derived from LEDs, which is mainly used in aesthetic medicine, depending on the radiated wavelength and angle of incidence, exerts an effect on tissues locally and has an ability to penetrate into their interior [24]. The abovementioned process of action confirms the results achieved in the authors' own research, which are undoubtedly influenced by the factor in question at the site of pain and an adequate penetration into painful tissue, which certainly leads to an analgesic effect. Simpson observed that near-infrared LEDs cause the deepest tissue penetration of visible wavelengths and are therefore used for targeted therapy in subcutaneous structures and fibroblasts [25]. There are many studies on the use of red LEDs in various diseases, including wound healing, precancerous treatment, warts, and prevention of oral mucositis. It has been observed that IR LED infrared light therapy is able to penetrate the skin and provide a therapeutic stimulus between 5 and 10 mm [26]. The authors' research and observations inferred a confirmation of the beneficial effect of the discussed method on the human body. At the same time, they proved an improvement in the analgesic effect after its use. Arneja, in contrast, used an electromagnetic field in the treatment of chronic spinal pain in patients with degenerative disease. These studies indicate the safety and effectiveness of the method and its high relevance in clinical trials [27]. Iannitti, in his clinical studies with the use of pulsed electromagnetic field among elderly people, obtained a beneficial effect of the therapy conducted in the form of reduction of pain and stiffness of joints, as well as improvement of physical fitness of the examined population [28]. These studies show how important the therapeutic factor is in the use of electromagnetic fields in therapy. The author's research also obtained an analgesic effect at the same level of significance. Nelson, on the other hand, found that noninvasive electromagnetic field therapy leads to a rapid and significant reduction of pain in the early stages of osteoarthritis of the knee joint. An analgesic effect was achieved in the study group composed of 34 patients at the level of *p* < 0.001 [29]. The analgesic effect of slow alternating electromagnetic fields with low frequency and magnetic induction is particularly important in the treatment of patients with pain. According to Wheeler, discomfort in musculoskeletal and fascial structures affects approximately 85% of people who are struggling with posttraumatic pain. Additionally, more than 90% of people are reporting to their doctor regarding pain in the course of other diseases. Components of these ailments may also be found in 55% of patients suffering from head and neck pain [30].

Cryotherapy, as one of the physical treatments with a wide range of applications, also reached an enormous interest among researchers and authors of many publications. It confirms the author's results and the beneficial effects of this type of treatment in the analgesic direction. Lateef et al., in their research, focused on the assessment of the effectiveness of the Hilotherm cooling system in reducing pain and postoperative oedema in patients after maxillofacial trauma and orthognathic surgeries. The study was conducted in 34 patients with these described symptoms. After the examination, analgesic (*p* < 0,01) and antioedematous (*p* < 0,01) effects were significantly better in the study group with the use of a cooling agent than in the control group not covered by the therapy. Additionally, patient satisfaction with the results of the postoperative treatment was noted [31]. The author's research in the group of patients covered by the local cryotherapy treatment also presented an analgesic effect, although, in the author's comparison, MLT yielded better results than CT. This may be due to a deeper local effect and the simultaneous use of both factors in a single treatment. The light of an adjusted length used concomitantly with electromagnetic field penetrates deeper than if used independently. Such a synergy of both the discussed factors is more beneficial from a therapeutic point of view. Chou and Liu, on the other hand, in their research, compared the efficacy between cold compresses (towel) and ice pack in postoperative care. The analysis included the temperature of the skin in the treated area, pain, and swelling. Both wet cryotherapy and dry cryotherapy effectively reduced postoperative discomfort. Cryotherapy, in the form of a cold compress, was more effective in reducing the subjective discomfort caused by surgery. However, cryotherapy with ice packs was more successful in reducing the local temperature in some areas after surgery [32]. Both the subjective feelings of a patient and the temperature drop of the skin layers in the area undergoing treatment are particularly important in the treatment of the patient, especially when oedema and pain are reported in a given case. The abovementioned studies are in line with the author's findings. Filho et al. reported that, among 14 patients after the extraction of defective third mandibular molars, cryotherapy was performed only on one side, while on the other side, the patient did not receive a cooling stimulus. The authors conducted clinical trials to determine the analgesic effect of the procedure, as well as to measure swelling before surgery, directly after the procedure and 24 and 48 hours after surgery. Cryotherapy was effective in reducing both the swelling and the level of pain experienced by the patients [33]. In our studies, among patients from the cryotherapy group, no oedema was observed. Moreover, taking into account the level of pain, a statistically significant decrease of values was revealed after the treatment. Similar results have been obtained by other authors conducting research in this area. Hirvonen et al. examined 60 patients with active seropositive arthritis by treating them with systemic cryotherapy, local cryotherapy, and cold compresses. After the application of these therapeutic factors, the pain was reduced in all groups, but with the best effect in a systemic cryotherapy group. It is also significant that no severe or persistent adverse effects were detected in the studied population [34]. The authors' comparative analysis demonstrate that the approximate values of temperature at the level of −110°C used in other experiments brought better results than the temperature conditions used in the authors' own study, i.e., −60°C. Higher values of the CT temperature used in the author's study stem from the fact that the treated area, i.e., the temporomandibular joint, was small. Nevertheless, an improvement after its use was obtained anyway. This is a peculiar feature of the procedure described because the TMJ area and the adjacent structures are very sensitive to external stimuli. Furthermore, in the authors' results, none of the studied patients reported any adverse effects from the therapy. Saito et al. evaluated whether there is an analgesic effect of cryotherapy after total hip arthroplasty (THA). In the group of patients who experienced THA and were treated with low temperatures, the pain after the procedure was much weaker. Moreover, the postoperative use of painkillers in the cryotherapy group was much lower than in the control group without the use of cold therapy. The results of this study confirm the potential benefit of this factor, which results in pain reduction during postoperative recovery of patients undergoing THA [35].

In another research, Więckiewicz et al. emphasised an especially important aspect in discussing the pain experience of the masticatory organ and the mental state of the patient. The analysis of many studies in these subjects indicated a correlation between masticatory pain and mental state changes [36]. This is a paramount aspect that, beyond any doubt, should be taken into account when defining a patient's eligibility for treatment, as well as during the therapy itself. This will allow for a multidirectional treatment and also covering the patient with psychological care. Rymaszewska et al. conducted research in this direction, assessing the impact of cryotherapy on psyche and mood disorders. In a group of 23 patients in the hospital suffering from depression, cold therapy was applied for 2 weeks. Antidepressants were not terminated. Symptoms were assessed at the beginning and end of the treatment using the 21-point Hamilton Depression Assessment Scale (HDRS). It was proved that the total HDRS score for each patient after cryotherapy was lower than the baseline score and was statistically significant. This indicates that cryotherapy had a positive effect on all symptoms, except for mood swings during the day and night. If research in this direction is extended, cryotherapy may become a supporting treatment for depression [37]. Symptoms subjected to the greatest improvement were anxiety and hyperactivity (90% of respondents), with both these factors certainly stimulating the development of TMD.

Through personal and other authors' research, it should be emphasised that the treatment of patients with the abovedescribed disorder also includes physiotherapy and physical therapy. Positive therapeutic effects are achieved by using laser therapy, heat therapy, light therapy, electrotherapy, electromagnetic field, manual therapy, proprioceptive neuromuscular traction, kinesitherapy, relaxation techniques, autogenous training, and biofeedback to change impaired behaviour [38]. Hals et al. consider that such a group of patients is often referred to as ‘difficult' because few health professionals believe they are able to help individually; therefore, it is essential to put these patients under the care of a multidisciplinary team that will guide the entire treatment process at each level [39].

## 6. Conclusions

1. Treatment with chosen MLT and CT physiotherapeutic methods, based on the analgesic effect in the course of TMD, brings a significant improvement in the subjective pain sensation determined on the VAS scale
2. The applied therapeutic methods caused a decreasing tendency of pain during individual mandibular movements, and making a comparison between them allowed us to observe statistically significant differences in favour of the MLT
3. The usage of selected physical factors and their beneficial effect on pain symptoms during the mandibular movements is an important aspect of everyday life and in the professional functioning of patients
4. The inclusion of therapeutic methods can lead to increased satisfaction and patient comfort when undertaking TMJ manual therapy as well as long-lasting dental procedures

## Figures and Tables

**Table 1:** Comparison of pain parameters on the VAS scale during the daily activity of the mandible before and after the therapy in each group (*n* = 30).

Therapy	Parameter	Mean	SD	Min	Max	*p*
MLT	Pain before	3.57	1.85	1.00	7.00	<0.001^*∗*^
	Pain after	5.83	1.72	2.00	9.00	

CT	Pain after	4.03	1.50	1.00	7.00	0.001^*∗*^
	Pain before	4.97	1.59	2.00	8.00	

^*∗*^ *n,* number of patients; SD, standard deviation; Min, minimum; Max, maximum. ^*∗*^Parameter statistically significant (Wilcoxon test).

**Table 2:** Results of the regression model estimation

	Estimate	Std. Error	*t* value	*p*
Intercept	−1.684	0.665	−2.534	0.014
MLT group	−1.306	0.272	−4.801	<0.001^*∗*^
Sex	0.219	0.284	0.772	0.443
Age	0.024	0.023	1.032	0.307

*Note*. A negative value means a greater pain reduction.

## Data Availability

The datasets used to support the findings of this study are available from the corresponding author upon request.
